# Molecular evidence of the hybrid origin of *Cryptocoryne* ×*purpurea* Ridl. nothovar. *purpurea* (Araceae)

**DOI:** 10.1371/journal.pone.0239499

**Published:** 2021-01-21

**Authors:** Rusly Rosazlina, Niels Jacobsen, Marian Ørgaard, Ahmad Sofiman Othman

**Affiliations:** 1 School of Biological Sciences, University Sains Malaysia, Minden, Penang, Malaysia; 2 Department of Plant and Environmental Sciences, Section of Organismal Biology, Faculty of Science, University of Copenhagen, Frederiksberg C, Copenhagen, Denmark; National Cheng Kung University, TAIWAN

## Abstract

Natural hybridization has been considered a source of taxonomic complexity in *Cryptocoryne*. A combined study of DNA sequencing data from the internal transcribed spacer (ITS) of nuclear ribosomal DNA and the *trn*K*-mat*K region of chloroplast DNA was used to identify the parents of *Cryptocoryne* putative hybrids from Peninsular Malaysia. Based on the intermediate morphology and sympatric distribution area, the plants were tentatively identified as the hybrid *Cryptocoryne* ×*purpurea* nothovar. *purpurea*. The plants were pollen sterile and had long been considered as hybrids, supposedly between two related and co-existing species, *C*. *cordata* var. *cordata* and *C*. *griffithii*. The status of *C*. ×*purpurea* nothovar. *purpurea* was independently confirmed by the presence of an additive ITS sequence pattern from these two parental species in hybrid individuals. An analysis of the chloroplast *trn*K*-mat*K sequences showed that the hybridization is bidirectional with the putative hybrids sharing identical sequences from *C*. *cordata* var. *cordata* and *C*. *griffithii*, indicating that both putative parental species had been the maternal parent in different accessions.

## Introduction

Natural interspecific hybridization has been demonstrated to be an important force in forming new species [1, 2] and plays a crucial role in plant evolution and diversification [3, 4]. The occurrence of natural hybridization between different species, however, is not universal but is concentrated among a limited fraction of plant families and genera [5]. Natural hybridization has been suggested to occur frequently in *Cryptocoryne*. The genus can be seen as having multiple populations in various river systems and hybridization can be an evolutionary driving force that constantly creates new genotypes that are spread across the ever-changing river systems [6–10]. Most recently, 65 species, 19 varieties and 15 named interspecific hybrids have been recognized [9–16]. Recognizing the *Cryptocoryne* hybrids began in the 1970s [17–19] and two of the *Cryptocoryne* hybrids have been recognized (as species) for more than 100 years. However, not until after 1975 was it realized and accepted that some of the plants were probably interspecific hybrids [17]. The uncertain status and tendency of *Cryptocoryne* to hybridize naturally may create more complexity in terms of taxonomic studies and classification. The natural *Cryptocoryne* hybrids have previously been reported in Peninsular Malaysia [6, 10, 17], Sri Lanka [19–21], Thailand and Lao P. D. R. [7, 18], Singapore [22], Sarawak [23], Kalimantan [14, 15, 24, 25] and Sumatera [13], with an overview presented by Jacobsen et al. [7]. Even though *Cryptocoryne* hybrids have greatly reduced fertility, the hybrids are highly successful due to the proliferous propagation by numerous, long, subterranean stolons, resulting in very large stands of hybrid plants, easily detectable in nature.

*Cryptocoryne* ×*purpurea* Ridl. nothovar. *purpurea* is a natural interspecific hybrid, which can be found in Peninsular Malaysia. Another natural interspecific hybrid, *C*. ×*purpurea* northovar. *borneoensis* can only be found in Borneo [24]. This Borneo hybrid differs from the Peninsular Malaysia hybrid by having a limb with a pronounced collar, a purple zone in the upper part of the kettle and a purple coloured appendix of the spadix. Additionally, the Bornean hybrid has a different chromosome number from the hybrid in Peninsular Malaysia and the former was suggested to have slightly different putative parents than the hybrid present in Peninsular Malaysia. According to Othman et al. [6], *Cryptocoryne* ×*purpurea* nothovar. *purpurea* was first collected from Kota Tinggi, Johor by Ridley in 1892, was cultivated in the Botanical Garden in Singapore and was shipped to Europe in 1898. It flowered at Kew and was pictured as *C*. *griffithii* Schott in the *Botanical Magazine* in 1900 (t 7719). In 1904, Ridley described this plant as a new species, named as *C*. *purpurea*. It was cultivated widely as an aquarium plant in Europe in the following years, although it almost disappeared towards the end of the century.

Based on the low pollen fertility [17], it was suggested that *C*. ×*purpurea* nothovar. *purpurea* was a hybrid of *C*. *cordata* Griff. var. *cordata* and *C*. *griffithii* Schott based on the coherence of morphological characteristics (broad collar zone–*C*. *cordata* var. *cordata*, and purple, rough spathe limb–*C*. *griffithii*) [6]. de Wit [26] gave a comprehensive explanation of the differences between *C*. *griffithii*, *C*. *cordata* var. *cordata* and *C*. ×*purpurea* nothovar. *purpurea*. Evidence of this morphological assumption gained support over the years and it is now generally accepted as a hybrid between the diploid *C*. *cordata* var. *cordata* and *C*. *griffithii*; moreover, both parents and the putative hybrid are found in the same region [6, 7].

Several molecular studies on the genus have been performed. One of the first molecular works focused on the phylogenetics of the genus, using nuclear and chloroplast DNAs [27]. The phylogeny of nuclear DNA suggests that *C*. ×*purpurea* nothovar. *purpurea* is closely associated with *C*. *cordata* var. *cordata* in contrast with the chloroplast DNA phylogeny, placing *C*. ×*purpurea* nothovar. *purpurea* in close association with *C*. *griffithii*. Although only one individual for each species was used, the results suggest that *C*. *griffithii* is the putative maternal parent and *C*. *cordata* var. *cordata* is the other putative parent in the studied material. In another study, random markers were used in the genetic studies of *C*. ×*purpurea* nothovar. *purpurea* from locations in the Tasik Bera (Bera Lake) system in Peninsular Malaysia which indicated the presence of two distinct genetic groups, suggesting that two independent hybridization events or bidirectional hybridization had taken place [28]. Ipor et al. [29] used random markers to genotype the different *C*. ×*purpurea* hybrids from Peninsular Malaysia, Sarawak and southern Kalimantan. Although the samples from Peninsular Malaysia and Borneo are treated as *C*. ×*purpurea* in a broad sense, their genetic results clearly separate the plants from Peninsular Malaysia and Borneo into two distinct clusters, supporting their different origins, i.e., *C*. ×*purpurea* nothovar. *purpurea*, 2*n* = 34 (*C*. *cordata* var. *cordata*, 2*n* = 34 × *C*. *griffithii*, 2*n* = 34) [17, 30], *C*. ×*purpurea* nothovar. *borneoensis*, 2*n* = 51 (*C*. *cordata* var. *grabowskii* (Engl.) N. Jacobsen, 2*n* = 68 × *C*. *griffithii*, 2*n* = 34) from southern Kalimantan [24]; the plants from Sungai Stungkor, Lundu, Sarawak, were later described as a separate pentaploid hybrid *C*. ×*batangkayanensis* Ipor et al. [23] with 2*n* = 85 between *C*. *cordata* var. *grabowskii* (2*n* = 68. unreduced) and *C*. *ferruginea* Engl. var. *ferruginea* (2*n* = 34) [23].

In a study on artificial hybridization between species of *Cryptocoryne* from Peninsular Malaysia, evidence indicated that *C*. ×*purpurea* nothovar. *purpurea* was a natural hybrid between *C*. *cordata* var. *cordata* and *C*. *griffithii* [8]. Verifying the hybrid origins of taxa in question is valuable in terms of studies on taxonomy, evolution and conservation. If parental species overlap at multiple locations across the geographical distribution, hybrids may emerge independently from hybridization of local parental genotype populations and may exhibit obvious morphological differences [31]. Therefore, different hybrid taxa with a slight morphological dissimilarity may have evolved as a result of hybridization of the same parental species at separate locations. In the present study, we compared two diverse *C*. ×*purpurea* nothovar. *purpurea* populations in streams only 2–3 km apart, which differed in shape, surface structure and colour of the spathe limb, representing two hybridization events with different parental genotypes in the state of Malacca [7]. Inferring the origins of such hybrid taxa based on morphology alone may, therefore, be difficult because morphologically similar hybrids can arise from the hybridization of different populations of the same parental species or be influenced by environmental conditions, which can be unreliable and misleading. In such cases, molecular means have been proven successful in identifying hybrid genotypes and determining the origins of various hybrid taxa [31–34].

Combined nuclear and plastid DNA markers provide potentially complementary evidence relating to a putative hybrid, allowing various questions to be investigated. To verify the hybrid origin of these intermediate individuals in the present study, a nuclear ribosomal DNA (nrDNA) internal transcribed spacer (ITS) region was first applied to examine the interspecific divergence between the putative parental species and assess the hybrid origin of the intermediate individuals by the additive patterns of both parental species. Secondly, the chloroplast *trn*K*-mat*K region was applied due to its success in evaluating interspecific variation in most angiosperms, and also its use in identifying the maternal origin of hybrids.

## Materials and methods

### Plant material

The individuals of the putative hybrid *C*. ×*purpurea* nothovar. *purpurea* and the presumed parental species *C*. *cordata* var. *cordata* and *C*. *griffithii* were collected from different locations, in order to detect the potential intraspecific sequence of polymorphism. The permission for field work was granted by the Forestry Department of Peninsular Malaysia. The Department of Orang Asli Development, Malaysia, have given sampling authorization to reach several locations in Tasik Bera. We sampled seven individuals of *C*. ×*purpurea* nothovar. *purpurea*, five individuals of *C*. *cordata* var. *cordata* and five individuals of *C*. *griffithii* respectively. For *C*. *griffithii* voucher number NJS 04–21, was presented to N. Jacobsen by Singapore Botanical Garden, from material grown in the garden while voucher number NJI 01–14 and NJS 04–21 were presented to N. Jacobsen by Oriental Aquarium, Singapore, Pte Ltd., from their nursery material. Since interspecific hybridization may be confounded by incomplete lineage sorting among closely related species, an additional *Cryptocoryne* species (*C*. *nurii* Furt. var. *nurii* and *C*. *schulzei* De Wit) was further examined. In addition, *C*. *nurii* var. *nurii* and *C*. *schulzei* were included in this study as their distribution was within the areas where the putative hybrids and presumed parental species are found. All accessions are summarized in Table 1.

**Table 1:** Taxa, localities, voucher numbers and list of abbreviations for sampled *Cryptocoryne* specimens.

Taxon	Locality	Voucher and collection number	List of abbreviations
*C*. ×*purpurea* Ridl. nothovar. *purpurea*	Kampung Pulau Semut, Masjid Tanah, Malacca ^a^	RR 11–06	MT
	Sungai Udang Recreational Forest, Malacca ^a^	RR 12–02	SU
	Pos Iskandar, Tasik Bera, Pahang ^a^	RR 13–07	PI
	Kampung Jelawat, Tasik Bera, Pahang ^a^	RR 13–08	KJ
	Paya Kelantong, Tasik Bera, Pahang ^a^	RR 13–09	PK
	Sungai Sedili Kechil, Kota Tinggi, Johor ^a^	RR 11–10	SED
	Kampung Sri Lukut, Kahang, Johor ^a^	RR 12–04	SL
*C*. *cordata* Griff. var. *cordata*	Gunung Arong, Mersing, Johor^a^	RR 12–05	GA
	Panti Bird Sanctuary, Kota Tinggi, Johor ^a^	RR 11–07	PAN
	Muadzam Shah, Pahang ^a^	RR 11–24	MU
	Sungai Tembangau, Tasik Bera, Pahang^a^	RR 10–03	ST
	Bukit Sedanan, Masjid Tanah, Malacca ^a^	RR 11–03	BS
*C*. *griffithii* Schott	Felda Nitar, Mersing, Johor^a^	RR 15–01	GF
	Kulai, Johor ^a^	NJM 01–3	KUL
	Bintan ^b^	NJI 01–14	BIN
	Singapore Botanical Garden ^c^	NJS 04–21	BOT
	Singapore (Oriental Aquarium) ^c^	NJS 01–16	SIN
*C*. *nurii* Furtado var. *nurii*	Kahang-Jemaluang, Mersing, Johor ^a^	RR 11–16	NKJ
	Sungai Kahang, Johor ^a^	RR 15–03	NSK
*C*. *schulzei* De Wit	Hutan Lipur Panti, Kota Tinggi, Johor ^a^	RR 11–21	SPAN
	Kahang-Jemaluang, Mersing, Johor ^a^	RR 11–17	SKJ

The latitude/longitude coordinates of the collection sites are not provided to ensure the protection of the species. RR are Rusly Rosazlina numbers and NJ are Niels Jacobsen numbers, deposited at USM. The first number after the initials represents the collection year and the second number is the running number.

^a^ Malaysia

^b^ Indonesia

^c^ Singapore.

**Fig 1:**
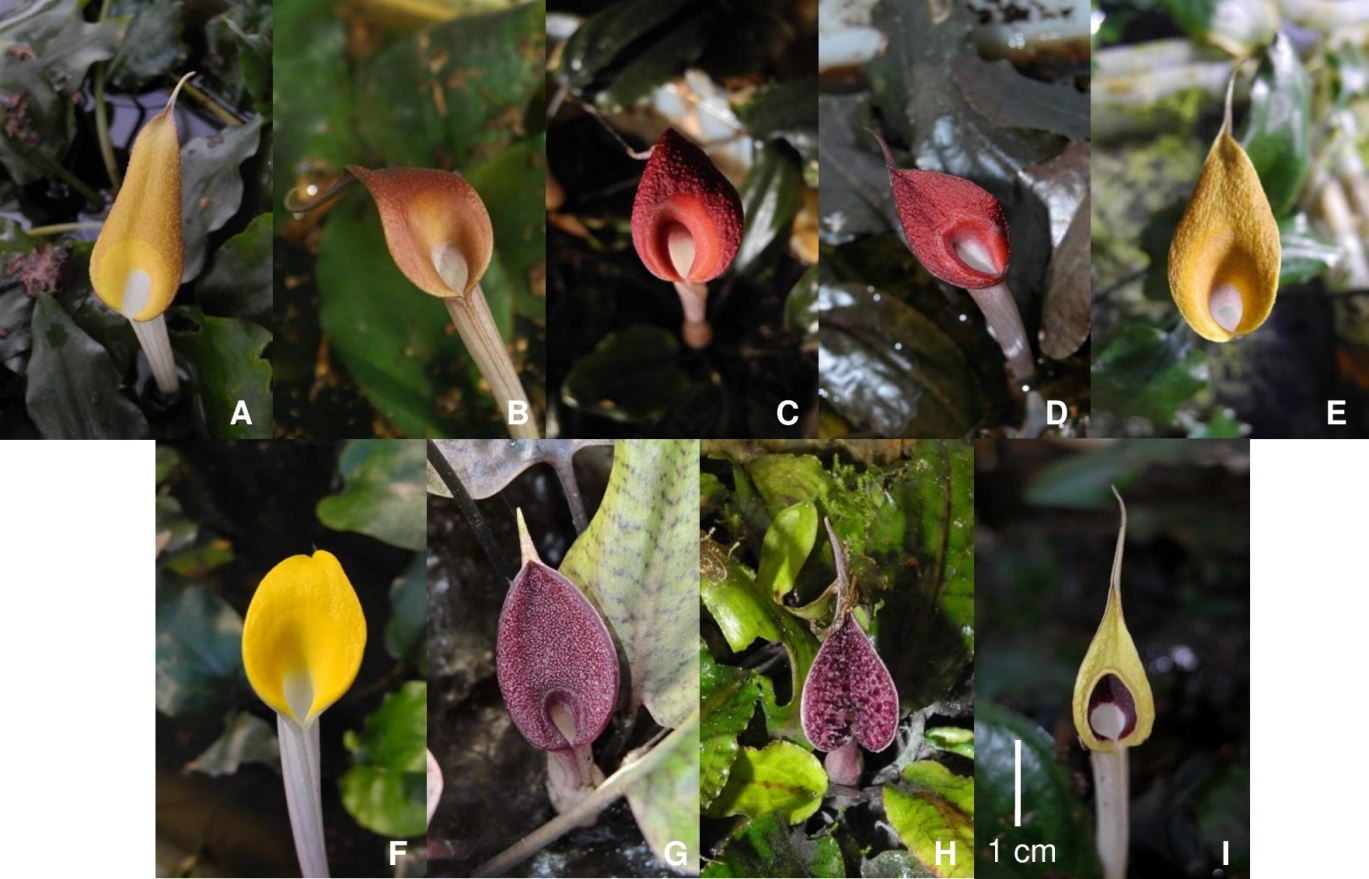
The spathe limbs of different accessions of *C*. ×*purpurea* nothovar. *purpurea* and the putative parental species. *C*. ×*purpurea* nothovar. *purpurea* (A, MT; B, SU; C, PI; D, SL; E, SED). *C*. *cordata* var. *cordata* (F, BS). *C*. *griffithii* (G, GF). *C*. *nurii* var. *nurii* (H, NSK). *C*. *schulzei* (I, SPAN). For the locality abbreviations, see Table 1.

Fig 1 shows the spathe limbs of different accessions of *C*. ×*purpurea* nothovar. *purpurea* and the putative parental species. RR voucher specimens have been deposited in the Herbarium Unit, Universiti Sains Malaysia, Penang (USMP) and NJ voucher specimens (and duplicates of most of the RR specimens) at the Botanical Museum, Copenhagen (C) (Natural History Museum of Denmark). Young leaves were cleaned with sterile distilled water before drying with silica gel. Upon completion of the drying process, samples were stored at -20°C before being used for DNA extraction.

### DNA extraction, PCR amplification and sequencing

The total genomic DNA was extracted from silica-dried leaf tissue using the CTAB protocol described by Doyle and Doyle [35]. The nrDNA ITS and cpDNA *trn*K*-mat*K regions were amplified using universal ITS1 and ITS4 primers [36] and *trn*K-3914F, *trn*K-2R primers [37] respectively. We obtained two newly designed primers for the *trn*K*-mat*K regions specifically for the *Cryptocoryne* species; *mat*KC-450F (5’-AGGGCAGAGTAGAGATGGATG-3’) and *mat*KC-537R (5’-TATCAGAATCCGGCAAATCG-3’). The PCR products were directly sequenced using PCR primers after purification with Gel Clean-up System (Promega Corporation, Madison, WI, USA). All sequencing reactions were carried out using the ABI 3700 DNA automated sequencer with the BigDye chemistry (Applied Biosystems, Foster City, CA, USA). The ITS PCR products which produced unreadable sequence data with superimposed peaks in the chromatograms, were further purified using the Wizard SV DNA Clean-Up System (Promega Corp., Madison, WI, USA) and then cloned to ensure representative amplification of the parental copies using the pGEM-T Easy-cloning Vector Kit (Promega Corp., Madison, WI, USA) and transformed into competent *Escherichia coli* JM109 at 42°C. The transformed bacteria were screened on solid LB media with 100 mg/mL ampicillin at 37°C overnight. The six positive clones with the correct size inserts were confirmed using colony PCR and subsequently sequenced using the ITS primers described previously. The possibility of artificial recombinants being produced under standard PCR conditions was also tested using ITS primers and a 1:1 mixture of *C*. *cordata* var. *cordata* (GA) and *C*. *griffithii* (KUL) DNA as a template. All of the sequences were deposited in GenBank with accession numbers KU196170-KU196197 and KU196237-KU196248.

### Sequence alignment and phylogenetic analyses

DNA sequences were assembled and aligned using (MEGA) 7.0 [38]. A most parsimonious (MP) unrooted tree was first built for ITS sequences of parents using PAUP* [39]. For this purpose, we conducted a heuristic search with starting trees obtained via stepwise addition with 100 iterations of the random addition sequence and the TBR branch-swapping option in PAUP*. All constants and variable (i.e., non-informative) characters were deleted. Only informative characters for parsimony were examined. The consistency index (CI) and the retention index (RI) were calculated, and the number of character changes were mapped on branches. Then, for each putative hybrid, we built an MP unrooted tree by combining its sequences with those of parents, using the same method of reconstruction and only parsimony-informative characters. Finally, an MP phylogenetic tree was built for all *trn*K*-mat*K sequences of putative hybrids, *C*. *cordata* var. *cordata*, *C*. *griffithii*, and for *C*. *nurii* var. *nurii* and *C*. *schulzei* as outgroups.

## Results

### Sequence analysis of the nrITS region

The nuclear ITS sequences had a total aligned length of 736 bp with 12 fixed nucleotide substitutions and indels indicated, which distinguished the *C*. *cordata* var. *cordata* sequences from the *C*. *griffithii* sequences at species level (Table 2). All fixed nucleotide substitutions with all clone accession numbers were summarized in S1 Table. In the case of the putative hybrid, all individuals exhibited chromatogram peak additivity at all these fixed sites. No intraspecific polymorphism was detected within each putative parental species. Among the 42 cloned sequences of *C*. ×*purpurea* nothovar. *purpurea*, 14 haplotypes (H1) were identical to *C*. *griffithii*; six haplotypes (H2) were identical to *C*. *cordata* var. *cordata*, and the remaining 22 cloned sequences (H3–H7) showed intermediate sequences between *C*. *cordata* var. *cordata* and *C*. *griffithii*. Of the six cloned sequences from the *C*. *cordata* var. *cordata* and *C*. *griffithii* template mixture, one was pure *C*. *griffithii* (H1), one was pure *C*. *cordata* var. *cordata* (H2), and the remaining four revealed intermediate sequences (H3). On the other hand, *C*. *nurii* var. *nurii* had ITS sequences different from those of *C*. ×*purpurea* nothovar. *purpurea* at six positions, which eliminated *C*. *nurii* var. *nurii* as a possible parent. However, *C*. *schulzei* had identical ITS profiles to those of *C*. *cordata* var. *cordata* and *C*. ×*purpurea* nothovar. *purpurea*. This additivity strongly supports the fact that *C*. ×*purpurea* nothovar. *purpurea* is the hybrid of *C*. *cordata* var. *cordata* and *C*. *griffithii*, although ITS data alone cannot reject the possibility of it being *C*. *griffithii* × *C*. *schulzei*.

**Table 2:** Variable nucleotide sites of ITS sequences’ comparison of the clones and the putative parental species.

Taxon		ITS Variable sites																	
		0	0	1	1	1	1	2	2	4	4	4	6	6	6	6	6	6	6
		4	4	4	4	8	9	3	5	2	5	7	0	2	4	6	6	6	8
		3	4	1	2	6	4	0	0	2	1	9	7	3	2	0	7	8	8
*C*. *griffithii*		C	T	A	A	G	A	G	A	T	C	T	C	A	A	C	−	−	T
*C*. *cordata* var. *cordata*		T	−	G	A	G	A	T	G	C	T	C	C	A	G	C	G	C	C
*C*. ×*purpurea* nothovar. *purpurea*	H1 (14)	C	T	A	A	G	A	G	A	T	C	T	C	A	A	C	−	−	T
*C*. ×*purpurea* nothovar. *purpurea*	H2 (6)	T	−	G	A	G	A	T	G	C	T	C	C	A	G	C	G	C	C
*C*. ×*purpurea* nothovar. *purpurea*	H3 (15)	C	T	G	A	G	A	T	G	C	T	C	C	A	G	C	G	C	C
*C*. ×*purpurea* nothovar. *purpurea*	H4 (4)	C	T	G	A	G	A	T	G	C	T	C	C	A	G	C	−	−	C
*C*. ×*purpurea* nothovar. *purpurea*	H5 (1)	C	T	A	A	G	A	G	A	T	T	T	C	A	G	C	−	−	T
*C*. ×*purpurea* nothovar. *purpurea*	H6 (1)	C	T	G	A	G	A	T	G	C	T	C	C	A	G	C	−	C	C
*C*. ×*purpurea* nothovar. *purpurea*	H7 (1)	T	−	G	A	G	A	T	G	C	T	C	C	A	G	C	−	−	C
DNA mixture	H1 (1)	C	T	A	A	G	A	G	A	T	C	T	C	A	A	C	−	−	T
DNA mixture	H2 (1)	T	−	G	A	G	A	T	G	C	T	C	C	A	G	C	G	C	C
DNA mixture	H3 (4)	C	T	G	A	G	A	T	G	C	T	C	C	A	G	C	G	C	C
*C*. *schulzei*		T	−	G	A	G	A	T	G	C	T	C	C	A	G	C	G	C	C
*C*. *nurii* var. *nurii*		C	T	A	G	C	G	G	A	T	C	T	A	G	A	G	−	−	T

The numbers represent the positions of the variable sites. Seven haplotypes (H1-H7) with individual numbers are found in *C*. ×*purpurea* nothovar. *purpurea*. The DNA mixture of *C*. *griffithii*; KUL and *C*. *cordata* var. *cordata*; GA. “−” denotes a gap.

**Fig 2:**
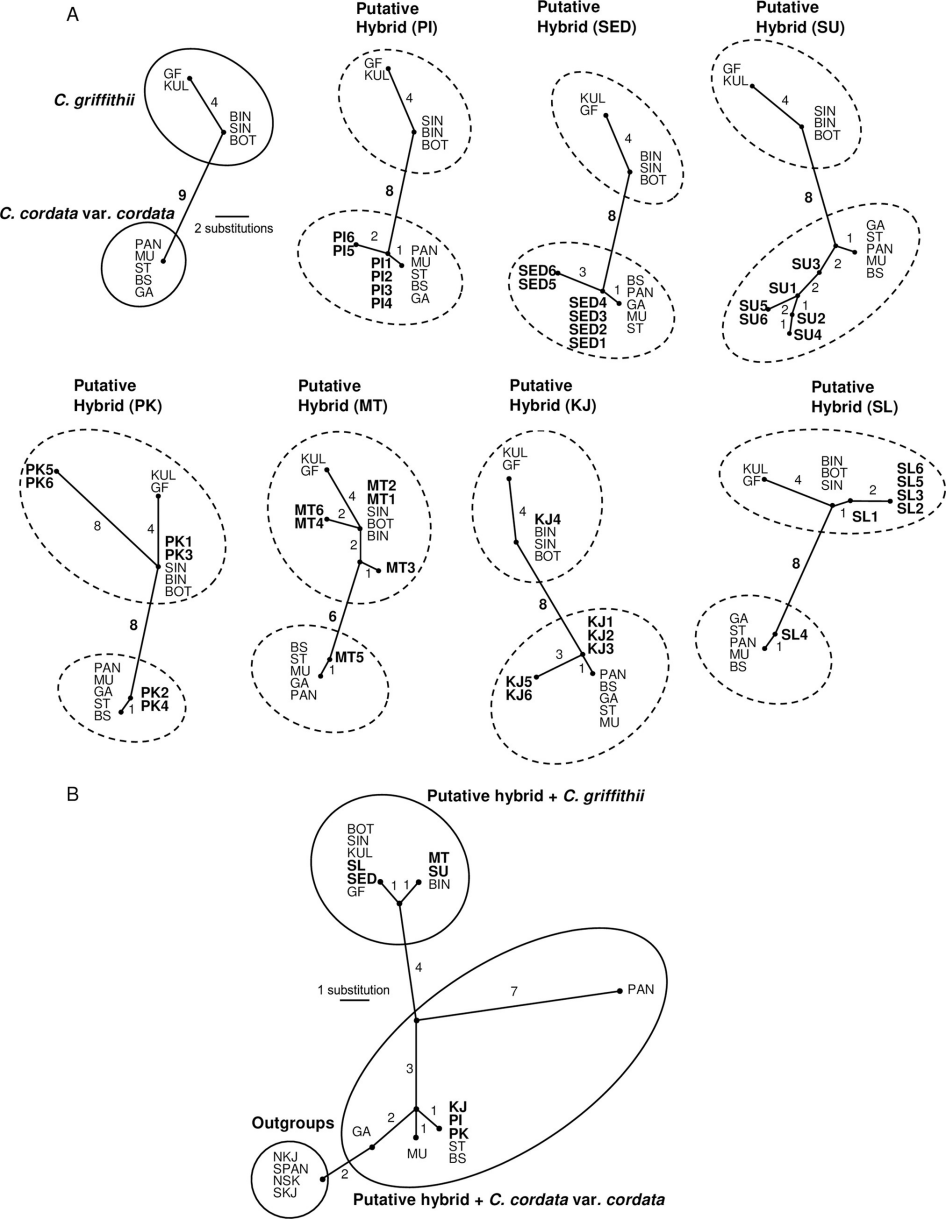
MP unrooted tree based on equal weighting of each character according to ITS (A) and *trn*K-*mat*K (B) sequences. Clone accession numbers were included in the ITS tree as per the S1 Table. The numbers close to the connecting lines denote mutational steps.

MP analysis based on equal weighting of each character, yielded one tree of 12 steps, with a CI of 1 and RI of 1. Fig 2A shows this tree in which the two putative parents, *C*. *griffithii* and *C*. *cordata* var. *cordata*, form two distinct groups that differ from one other by nine unique substitutions (= fixed differences). An analysis of the putative clonal hybrid sequences showed that of the four putative hybrids (PK, MT, KJ and SL), we identified two groups of sequences corresponding to *C*. *cordata* var. *cordata* and *C*. *griffithii*. These two groups differed from one other by six to eight unique substitutions. For putative hybrids PI, SED and SU, only sequences similar to *C*. *cordata* var. *cordata* were detected from the six sequenced clones.

### Sequence analysis of the cpDNA region

The aligned length of the chloroplast *trn*K-*mat*K region was 1980 bp across the whole dataset. An alignment of consensus nucleotide sequences from all samples varied at 20 sites (Table 3). In relation to these positions, four substitutions and six single-base pair indels distinguished the *C*. *cordata* var. *cordata* sequences from the *C*. *griffithii* sequences. No variation was identified in species sampled from the same location. The comparison showed that the putative hybrid samples from PI, KJ and PK had sequences identical to *C*. *cordata* var. *cordata* of ST and BS while SL and SED had sequences identical to *C*. *griffithii* (GF, KUL, BOT, SIN). The *C*. ×*purpurea* nothovar. *purpurea* accessions from MT and SU had sequences identical to *C*. *griffithii* (BIN) at one nucleotide position. The *trn*K*-mat*K sequences of *C*. *cordata* var. *cordata* (PAN) differed by three substitutions and *C*. *cordata* var. *cordata* (GA) differed by two substitutions when compared to other *C*. *cordata* var. *cordata* accessions. The nucleotide composition for *C*. *nurii* var. *nurii* was identical to *C*. *schulzei* and dissimilar to that of *C*. ×*purpurea* nothovar. *purpurea* at two nucleotide positions, rendering it unlikely that both *C*. *nurii* var. *nurii* and *C*. *schulzei* can be parents to *C*. ×*purpurea* nothovar. *purpurea*.

**Table 3:** Variable nucleotide sites of *trn*K*-mat*K sequences’ comparison of *C*. ×*purpurea* nothovar. *purpurea* and the putative maternal parents.

Taxon	Variable sites																			
	0	0	0	0	0	0	0	0	0	0	0	0	0	0	1	1	1	1	1	1
	1	1	1	1	1	1	1	2	2	3	4	4	5	5	0	0	1	1	2	3
	4	6	6	6	6	6	6	4	6	6	1	9	4	5	5	8	7	8	3	8
	8	4	5	6	7	8	9	1	3	7	0	4	3	0	0	9	6	2	9	9
*C*. ×*purpurea* nothovar. *purpurea*; PI	G	−	−	−	−	−	−	T	T	G	T	T	T	T	G	A	T	T	C	T
*C*. ×*purpurea* nothovar. *purpurea*; KJ	G	−	−	−	−	−	−	T	T	G	T	T	T	T	G	A	T	T	C	T
*C*. ×*purpurea* nothovar. *purpurea*; PK	G	−	−	−	−	−	−	T	T	G	T	T	T	T	G	A	T	T	C	T
*C*. *cordata* var. *cordata*; ST	G	−	−	−	−	−	−	T	T	G	T	T	T	T	G	A	T	T	C	T
*C*. *cordata* var. *cordata*; BS	G	−	−	−	−	−	−	T	T	G	T	T	T	T	G	A	T	T	C	T
*C*. *cordata* var. *cordata*; MU	G	−	−	−	−	−	−	T	T	G	T	T	G	T	G	A	T	T	C	T
*C*. *cordata* var. *cordata*; GA	G	−	−	−	−	−	−	T	T	T	T	T	G	T	G	A	T	T	C	A
*C*. *cordata* var. *cordata*; PAN	G	−	−	−	−	−	−	T	T	G	C	T	G	T	A	A	G	T	C	T
*C*. ×*purpurea* nothovar. *purpurea*; SL	G	C	T	G	T	A	T	T	G	G	C	T	G	G	A	G	G	C	A	T
*C*. ×*purpurea* nothovar. *purpurea*; SED	G	C	T	G	T	A	T	T	G	G	C	T	G	G	A	G	G	C	A	T
*C*. *griffithii*; GF	G	C	T	G	T	A	T	T	G	G	C	T	G	G	A	G	G	C	A	T
*C*. *griffithii*; KUL	G	C	T	G	T	A	T	T	G	G	C	T	G	G	A	G	G	C	A	T
*C*. *griffithii*; BOT	G	C	T	G	T	A	T	T	G	G	C	T	G	G	A	G	G	C	A	T
*C*. *griffithii*; SIN	G	C	T	G	T	A	T	T	G	G	C	T	G	G	A	G	G	C	A	T
*C*. ×*purpurea* nothovar. *purpurea*; MT	A	C	T	G	T	A	T	T	G	G	C	T	G	T	A	G	G	C	A	T
*C*. ×*purpurea* nothovar. *purpurea*; SU	A	C	T	G	T	A	T	T	G	G	C	T	G	T	A	G	G	C	A	T
*C*. *griffithii*; BIN	A	C	T	G	T	A	T	T	G	G	C	T	G	T	A	G	G	C	A	T
*C*. *schulzei*; SPAN	G	−	−	−	−	−	−	G	T	T	T	A	G	T	G	A	T	T	C	A
*C*. *schulzei*; SKJ	G	−	−	−	−	−	−	G	T	T	T	A	G	T	G	A	T	T	C	A
*C*. *nurii* var. *nurii*; NKJ	G	−	−	−	−	−	−	G	T	T	T	A	G	T	G	A	T	T	C	A
*C*. *nurii* var. *nurii*; NSK	G	−	−	−	−	−	−	G	T	T	T	A	G	T	G	A	T	T	C	A

The numbers represent the positions of variable sites. “−” denotes a gap.

A phylogenetic analysis with the MP algorithm yielded one MP tree of 20 steps, with a CI of 1 and RI of 1. In this tree (Fig 2B), the ingroup and *C*. *griffithii* are each monophyletic, while *C*. *cordata* var. *cordata* is not monophyletic because outgroups are nested within. When the *trn*K*-mat*K sequences of the putative hybrids are added to the dataset, the resulting MP tree shows that the maternal origin of the *trn*K*-mat*K sequences of the putative hybrids KJ, PI and PK is *C*. *cordata* var. *cordata*, whereas *C*. *griffithii* is the parent of the *trn*K*-mat*K sequences of the putative hybrids SL, SED, MT and SU.

## Discussion

The nuclear genes are biparentally inherited, and the hybrids should possess both divergent copies of their putative parents [33, 34]. The nuclear ITS sequences of *C*. ×*purpurea* nothovar. *purpurea* showed nucleotide polymorphism at some sites, whereas those of *C*. *cordata* var. *cordata* and *C*. *griffithii* did not (Table 2); the data from the *C*. ×*purpurea* nothovar. *purpurea* accessions exhibited polymorphism patterns which are consistently formed by additive sequences derived from two hypothesized parental species. The present ITS data revealed that *C*. ×*purpurea* nothovar. *purpurea* possessed hybrid genotypes, having both ITS haplotypes of parental *C*. *cordata* var. *cordata* and *C*. *griffithii*, therefore, suggesting that *C*. ×*purpurea* nothovar. *purpurea* is a natural hybrid of these two species. The results also showed that 22 (52.4%) out of the 42 cloned nrITS sequences from *C*. ×*purpurea* nothovar. *purpurea* were intermediate/chimeric (recombinations of parental sequence haplotypes (H3-H7)). In diploid hybrids, the co-occurrence of parental nrITS sequences is rarely maintained in subsequent generations due to concerted evolution; if concerted evolution is incomplete, then sampled genes may represent a mixture of non-homogenized paralogous sequences [40]. The effects of concerted evolution commonly occur after meiosis (sexual reproduction) but only in fertile plants [40–42]. However, Jacobsen [17] reported that the pollen of *C*. ×*purpurea* nothovar. *purpurea* is completely sterile which was also the case in the samples being investigated. Cytological analysis shows that *C*. ×*purpurea* nothovar. *purpurea* shares the same diploid chromosome number 2*n* = 34 as *C*. *cordata* var. *cordata* and *C*. *griffithii* [17, 30], which rules out the possibility of *C*. ×*purpurea* nothovar. *purpurea* being a sterile polyploid. Since *C*. ×*purpurea* nothovar. *purpurea* is sterile, all *C*. ×*purpurea* nothovar. *purpurea* individuals are assumed to represent an F_1_ generation. Therefore, F_1_ individuals should possess both parental ITS alleles without the impact of concerted evolution.

One explanation for the origin of such a unique allele in *C*. ×*purpurea* nothovar. *purpurea* may be the result of PCR-mediated recombination, a process of *in vitro* chimera formation from related DNA template sequences coamplified in a single PCR reaction [43]. PCR-mediated recombination results from either polymerase template switching during PCR or annealing of prematurely terminated products to non-homologous templates [43, 44]. This phenomenon has been well characterized from other hybrid plant species including *Potamogeton intortusifolius* [45], *Malus toringoides* [46] and *Aster chusanensis* [47]. In this study, the detection of chimeric haplotypes (H3) from an artificial DNA template (1:1 DNA mixture of *C*. *cordata* var. *cordata* and *C*. *griffithii*) may suggest that PCR-mediated recombination could be an explanation for the origin of chimeric nrITS PCR amplicons in *C*. ×*purpurea* nothovar. *purpurea*. Moreover, the chimeric haplotypes are unequally distributed in the nucleotide positions which indicates the process of recombination occurring in a non-random order during PCR. Techniques involving passing traditional PCR and cloning processes are necessary for further examination of the structure and evolution of nrITS sequences in *C*. ×*purpurea* nothovar. *purpurea*.

Due to the high sterility of *C*. ×*purpurea* nothovar. *purpurea*, derived from the low pollen stainability with cotton blue [13], it may be inferred that sterility is also prevalent of the female side. However, we cannot rule out that a backcrossing from one of the putative parents may take place, but we have not observed any cases where that might have occurred in the present context, or perhaps our material is too limited to draw such conclusions. In other cases, regarding the *Cryptocoryne* species from Sri Lanka [21] second or more generations have been reported and assumed, but here the hybrids showed a certain degree of fertility. In *C*. *crispatula* Engl. *s*.*l*. from Mainland Asia, multiple hybrids have been observed and there is some fertility in the hybrids; backcrosses and introgressions are also suggested to be highly likely [7].

The results showed that the ITS sequences of *C*. *cordata* var. *cordata* and *C*. *schulzei* are very similar, suggesting that the *C*. ×*purpurea* nothovar. *purpurea* populations examined are hybrids of *C*. *griffithii* and either *C*. *cordata* var. *cordata* or *C*. *schulzei*. The hybrid has been found to grow sympatrically with *C*. *griffithii* and *C*. *cordata* var. *cordata* in Malacca and S.W. Johor, but also with *C*. *schulzei* in S.E. Johor. *Cryptocoryne* species are mainly identified using floral characters, particularly the limb of the spathe. Although the colours of the limb of the spathe of *C*. ×*purpurea* nothovar. *purpurea* may vary, the surface structure shows intermediate characters between *C*. *cordata* var. *cordata* and *C*. *griffithii*, the broad collar zone being present in both *C*. ×*purpurea* nothovar. *purpurea* and *C*. *cordata* var. *cordata* and a rather rugose limb of the spathe, with a wide, pronounced collar is found in *C*. *griffithii*. *C*. *schulzei* does have a broad collar zone but has a strongly reflexed spathe limb [6]. A rough purple red limb of the spathe is characteristic of *C*. *griffithii* and resembles that of *C*. ×*purpurea* nothovar. *purpurea*; *C*. *nurii* var. *nurii* has a deep red to dark purple spathe with large irregular protuberances on the limb. In conclusion, with the joint examination of molecular and morphological datasets from the included accessions, it is unlikely that *C*. *schulzei* and *C*. *nurii* var. *nurii* are parents to *C*. ×*purpurea* nothovar. *purpurea*. In the aforementioned study on the artificial hybridization of species of *Cryptocoryne* from the Malay Peninsula, hybrids were produced between *C*. *cordata* var. *cordata* and *C*. *nurii* var. *nurii* which were unlike *C*. ×*purpurea* nothovar. *purpurea* [8]. Based on morphological characters, chromosome numbers and artificial hybridizations, two recent studies [9, 10] proposed that *C*. ×*decus-silvae* De Wit represents the hybrid *C*. *cordata* var. *cordata* **×** *C*. *nurii* var. *nurii*, *C*. ×*griffithiioides* N. Jacobsen represents the hybrid *C*. *griffithii* × *C*. *nurii* var. *nurii* and *C*. ×*schulzeioides* N. Jacobsen represents the hybrid *C*. *griffithii* × *C*. *schulzei*.

It is common to find more than one species of *Cryptocoryne* in southern Peninsular Malaysia (Pahang, Johor and Malacca) that share the same stream or river system. Suitable combinations of species coexist, resulting in hybridization conditions. The *trn*K*-mat*K sequences of the *C*. ×*purpurea* nothovar. *purpurea* accessions PI, KJ and PK from Pahang (all originating from the Tasik Bera) were identical to *C*. *cordata* var. *cordata* and highlighted this species as the maternal parent (Table 3, Fig 2B). Neither putative parent was found in the hybrid sampling lake, but *C*. *cordata* var. *cordata* (ST) was present in the nearby swamp. The origin of the Tasik Bera area dates back to only 4500 years B.P. [48]. Based on the explanation of Othman et al. [6], the main drainage of the Tasik Bera is now northward to Sungai Pahang (Pahang River), but there is still a small connection southward to the Sungai Palong/Sungai Muar, Johor that was formally the main run-off. This historical evidence provides the explanation as to how *C*. ×*purpurea* nothovar. *purpurea* has arisen as a hybrid of the more widespread *C*. *cordata* var. *cordata* and the southerly distributed *C*. *griffithii*, which has then spread along the west coast during the change in drainage systems. The *trn*K*-mat*K sequences of the *C*. ×*purpurea* nothovar. *purpurea* accessions from Johor (SL and SED) were identical to the *C*. *griffithii* populations from Johor (KUL and GF) and *C*. *griffithii* from Singapore (BOT and SIN). Currently it is known that the two putative parental species have both recently been found at the Sungai Sedili Kechil, Johor, and previous records have shown that *C*. *cordata* var. *cordata* and *C*. *griffithii* distribution overlapped in Johor [7, 49]. *C*. *griffithii* was also proposed as the maternal parent of the *C*. ×*purpurea* nothovar. *purpurea* populations from the Malacca region (MT and SU), viz. *C*. *griffithii* from BIN (Bintan, Indonesia). The other parental species *C*. *cordata* var. *cordata* (BS) were found within a distance of <40 km from all the hybrid locations in the Malacca region but *C*. *griffithii* has not been recorded in these locations recently. However, *C*. *griffithii* has previously been recorded as growing in several places in Malacca [6, 19]. The present results indicate that both *C*. *cordata* var. *cordata* and *C*. *griffithii* have served as the maternal donor and the different hybrid populations possess separate and independent origins. There was no distinct bias of maternal composition for either one of them and this suggests that natural hybridization between the two examined species is bidirectional.

The combined investigation of nuclear ribosomal DNA, viz. the ITS and the chloroplast DNA for the *trn*K*-mat*K region, provide compelling evidence for the natural hybridization of *C*. *cordata* var. *cordata* and *C*. *griffithii*. Molecular data support the hypothesis that the morphologically intermediate plants are hybrids which share *trn*K*-mat*K sequences identical to both *C*. *cordata* var. *cordata* and *C*. *griffithii*. As *C*. ×*purpurea* nothovar. *purpurea* is pollen sterile with the absence of meiotic recombination, the parental sequences of the nuclear and chloroplast markers may be retained in the vegetative progenies. This study provides substantial evidence for interspecific hybridizations in *Cryptocoryne*. It should be interesting to further investigate the population genetics, ploidy level and reproductive behaviour of the hybrids including the geographical distribution and the timing of hybridization events for a better understanding of the total extent of the hybridization process in *Cryptocoryne*.

## Supporting information

S1 Table Variable sites of ITS between the clone accession numbers and the putative parental species. (PDF)
