# Supplementary material for: Molecular evidence of the hybrid origin of Cryptocoryne ×purpurea Ridl. nothovar. purpurea (Araceae)
Source: PLoS One. 2021 Jan 21;16(1):e0239499. doi: 10.1371/journal.pone.0239499 (PMC7819605; doi:10.1371/journal.pone.0239499)
Supplement: S1 Table — (PDF) [file pone.0239499.s001.pdf]

S1 Table. Variable sites of ITS between the clone accession numbers and the putative parental species.

Taxon	Haplotype	Accession	Clone no	ITS Variable sites																	
				043	044	141	142	186	194	230	250	422	451	479	607	623	642	660	667	668	688
C. ×*purpurea* nothovar. *purpurea*	H1	MT	MT1	C	T	A	A	G	A	G	A	T	C	T	C	A	A	C	–	–	T
C. ×*purpurea* nothovar. *purpurea*	H1	MT	MT2	C	T	A	A	G	A	G	A	T	C	T	C	A	A	C	–	–	T
C. ×*purpurea* nothovar. *purpurea*	H1	MT	MT4	C	T	A	A	G	A	G	A	T	C	T	C	A	A	C	–	–	T
C. ×*purpurea* nothovar. *purpurea*	H1	MT	MT6	C	T	A	A	G	A	G	A	T	C	T	C	A	A	C	–	–	T
C. ×*purpurea* nothovar. *purpurea*	H1	KJ	KJ4	C	T	A	A	G	A	G	A	T	C	T	C	A	A	C	–	–	T
C. ×*purpurea* nothovar. *purpurea*	H1	PK	PK1	C	T	A	A	G	A	G	A	T	C	T	C	A	A	C	–	–	T
C. ×*purpurea* nothovar. *purpurea*	H1	PK	PK3	C	T	A	A	G	A	G	A	T	C	T	C	A	A	C	–	–	T
C. ×*purpurea* nothovar. *purpurea*	H1	PK	PK5	C	T	A	A	G	A	G	A	T	C	T	C	A	A	C	–	–	T
C. ×*purpurea* nothovar. *purpurea*	H1	PK	PK6	C	T	A	A	G	A	G	A	T	C	T	C	A	A	C	–	–	T
C. ×*purpurea* nothovar. *purpurea*	H1	SL	SL1	C	T	A	A	G	A	G	A	T	C	T	C	A	A	C	–	–	T
C. ×*purpurea* nothovar. *purpurea*	H1	SL	SL2	C	T	A	A	G	A	G	A	T	C	T	C	A	A	C	–	–	T
C. ×*purpurea* nothovar. *purpurea*	H1	SL	SL3	C	T	A	A	G	A	G	A	T	C	T	C	A	A	C	–	–	T
C. ×*purpurea* nothovar. *purpurea*	H1	SL	SL5	C	T	A	A	G	A	G	A	T	C	T	C	A	A	C	–	–	T
C. ×*purpurea* nothovar. *purpurea*	H1	SL	SL6	C	T	A	A	G	A	G	A	T	C	T	C	A	A	C	–	–	T
C. griffithii	H1	GF	*	C	T	A	A	G	A	G	A	T	C	T	C	A	A	C	–	–	T
C. griffithii	H1	KUL	*	C	T	A	A	G	A	G	A	T	C	T	C	A	A	C	–	–	T
C. griffithii	H1	BIN	*	C	T	A	A	G	A	G	A	T	C	T	C	A	A	C	–	–	T
C. griffithii	H1	BOT	*	C	T	A	A	G	A	G	A	T	C	T	C	A	A	C	–	–	T
C. griffithii	H1	SIN	*	C	T	A	A	G	A	G	A	T	C	T	C	A	A	C	–	–	T
C. ×*purpurea* nothovar. *purpurea*	H2	KJ	KJ5	T	–	G	A	G	A	T	G	C	T	C	C	A	G	C	G	C	C
C. ×*purpurea* nothovar. *purpurea*	H2	KJ	KJ6	T	–	G	A	G	A	T	G	C	T	C	C	A	G	C	G	C	C
C. ×*purpurea* nothovar. *purpurea*	H2	SU	SU3	T	–	G	A	G	A	T	G	C	T	C	C	A	G	C	G	C	C
C. ×*purpurea* nothovar. *purpurea*	H2	SU	SU4	T	–	G	A	G	A	T	G	C	T	C	C	A	G	C	G	C	C
C. ×*purpurea* nothovar. *purpurea*	H2	SU	SU5	T	–	G	A	G	A	T	G	C	T	C	C	A	G	C	G	C	C
C. ×*purpurea* nothovar. *purpurea*	H2	SU	SU6	T	–	G	A	G	A	T	G	C	T	C	C	A	G	C	G	C	C
C. cordata var. *cordata*	H2	GA	*	T	–	G	A	G	A	T	G	C	T	C	C	A	G	C	G	C	C
C. cordata var. *cordata*	H2	PAN	*	T	–	G	A	G	A	T	G	C	T	C	C	A	G	C	G	C	C

C. cordata var. *cordata*	H2	MU	*	T	–	G	A	G	A	T	G	C	T	C	C	A	G	C	G	C	C
C. cordata var. *cordata*	H2	ST	*	T	–	G	A	G	A	T	G	C	T	C	C	A	G	C	G	C	C
C. cordata var. *cordata*	H2	BS	*	T	–	G	A	G	A	T	G	C	T	C	C	A	G	C	G	C	C
C. ×*purpurea* nothovar. *purpurea*	H3	MT	MT5	C	T	G	A	G	A	T	G	C	T	C	C	A	G	C	G	C	C
C. ×*purpurea* nothovar. *purpurea*	H3	SU	SU1	C	T	G	A	G	A	T	G	C	T	C	C	A	G	C	G	C	C
C. ×*purpurea* nothovar. *purpurea*	H3	SU	SU2	C	T	G	A	G	A	T	G	C	T	C	C	A	G	C	G	C	C
C. ×*purpurea* nothovar. *purpurea*	H3	PI	PI4	C	T	G	A	G	A	T	G	C	T	C	C	A	G	C	G	C	C
C. ×*purpurea* nothovar. *purpurea*	H3	PI	PI5	C	T	G	A	G	A	T	G	C	T	C	C	A	G	C	G	C	C
C. ×*purpurea* nothovar. *purpurea*	H3	PI	PI6	C	T	G	A	G	A	T	G	C	T	C	C	A	G	C	G	C	C
C. ×*purpurea* nothovar. *purpurea*	H3	PK	PK2	C	T	G	A	G	A	T	G	C	T	C	C	A	G	C	G	C	C
C. ×*purpurea* nothovar. *purpurea*	H3	PK	PK4	C	T	G	A	G	A	T	G	C	T	C	C	A	G	C	G	C	C
C. ×*purpurea* nothovar. *purpurea*	H3	SL	SL4	C	T	G	A	G	A	T	G	C	T	C	C	A	G	C	G	C	C
C. ×*purpurea* nothovar. *purpurea*	H3	SED	SED1	C	T	G	A	G	A	T	G	C	T	C	C	A	G	C	G	C	C
C. ×*purpurea* nothovar. *purpurea*	H3	SED	SED2	C	T	G	A	G	A	T	G	C	T	C	C	A	G	C	G	C	C
C. ×*purpurea* nothovar. *purpurea*	H3	SED	SED3	C	T	G	A	G	A	T	G	C	T	C	C	A	G	C	G	C	C
C. ×*purpurea* nothovar. *purpurea*	H3	SED	SED4	C	T	G	A	G	A	T	G	C	T	C	C	A	G	C	G	C	C
C. ×*purpurea* nothovar. *purpurea*	H3	SED	SED5	C	T	G	A	G	A	T	G	C	T	C	C	A	G	C	G	C	C
C. ×*purpurea* nothovar. *purpurea*	H3	SED	SED6	C	T	G	A	G	A	T	G	C	T	C	C	A	G	C	G	C	C
C. ×*purpurea* nothovar. *purpurea*	H4	PI	PI1	C	T	G	A	G	A	T	G	C	T	C	C	A	G	C	–	–	C
C. ×*purpurea* nothovar. *purpurea*	H4	PI	PI3	C	T	G	A	G	A	T	G	C	T	C	C	A	G	C	–	–	C
C. ×*purpurea* nothovar. *purpurea*	H4	KJ	KJ1	C	T	G	A	G	A	T	G	C	T	C	C	A	G	C	–	–	C
C. ×*purpurea* nothovar. *purpurea*	H4	KJ	KJ2	C	T	G	A	G	A	T	G	C	T	C	C	A	G	C	–	–	C
C. ×*purpurea* nothovar. *purpurea*	H5	MT	MT3	C	T	A	A	G	A	G	A	T	T	T	C	A	G	C	–	–	T
C. ×*purpurea* nothovar. *purpurea*	H6	KJ	KJ3	C	T	G	A	G	A	T	G	C	T	C	C	A	G	C	–	C	C
C. ×*purpurea* nothovar. *purpurea*	H7	PI	PI2	T	–	G	A	G	A	T	G	C	T	C	C	A	G	C	–	–	C
DNA mixture	H1	§	MIX1	C	T	A	A	G	A	G	A	T	C	T	C	A	A	C	–	–	T
DNA mixture	H2	§	MIX5	T	–	G	A	G	A	T	G	C	T	C	C	A	G	C	G	C	C
DNA mixture	H3	§	MIX2	C	T	G	A	G	A	T	G	C	T	C	C	A	G	C	G	C	C
DNA mixture	H3	§	MIX3	C	T	G	A	G	A	T	G	C	T	C	C	A	G	C	G	C	C
DNA mixture	H3	§	MIX4	C	T	G	A	G	A	T	G	C	T	C	C	A	G	C	G	C	C
DNA mixture	H3	§	MIX6	C	T	G	A	G	A	T	G	C	T	C	C	A	G	C	G	C	C
C. schulzei		SPAN	*	T	–	G	A	G	A	T	G	C	T	C	C	A	G	C	G	C	C
C. schulzei		SKJ	*	T	–	G	A	G	A	T	G	C	T	C	C	A	G	C	G	C	C
C. nurii var. *nurii*		NKJ	*	C	T	A	G	C	G	G	A	T	C	T	A	G	A	G	–	–	T

C. nurii var. *nurii*		NSK	*	C	T	A	G	C	G	G	A	T	C	T	A	G	A	G	–	–	T

*****direct sequencing of PCR products; §genomic DNA mixture of *C. griffithii*; KUL and *C. cordata* var. *cordata*; GA. "–" denotes a gap.
